# Integrated genomic and DNA methylation analysis of patients with advanced non-small cell lung cancer with brain metastases

**DOI:** 10.1186/s13041-021-00886-4

**Published:** 2021-12-24

**Authors:** Yanjun Xu, Zhiyu Huang, Xiaoqing Yu, Kaiyan Chen, Yun Fan

**Affiliations:** Department of Medical Thoracic Oncology, The Cancer Hospital of the University of Chinese Academy of Sciences (Zhejiang Cancer Hospital), Institute of Basic Medicine and Cancer (IBMC), Chinese Academy of Sciences, No. 1 East Banshan Road, Gongshu District, Hangzhou, 310022 China

**Keywords:** Brain metastases, Predictive biomarker, DNA methylation, Somatic mutation, Advanced non-small cell lung cancer (advanced NSCLC)

## Abstract

**Background:**

Brain metastasis is a common and lethal complication of non-small cell lung cancer (NSCLC). It is mostly diagnosed only after symptoms develop, at which point very few treatment options are available. Therefore, patients who have an increased risk of developing brain metastasis need to be identified early. Our study aimed to identify genomic and epigenomic biomarkers for predicting brain metastasis risk in NSCLC patients.

**Methods:**

Paired primary lung tumor tissues and either brain metastatic tissues or cerebrospinal fluid (CSF) samples were collected from 29 patients with treatment-naïve advanced NSCLC with central nervous system (CNS) metastases. A control group comprising 31 patients with advanced NSCLC who died without ever developing CNS metastasis was also included. Somatic mutations and DNA methylation levels were examined through capture-based targeted sequencing with a 520-gene panel and targeted bisulfite sequencing with an 80,672 CpG panel.

**Results:**

Compared to primary lung lesions, brain metastatic tissues harbored numerous unique copy number variations. The tumor mutational burden was comparable between brain metastatic tissue (P = 0.168)/CSF (P = 0.445) and their paired primary lung tumor samples. Kelch-like ECH-associated protein (*KEAP1*) mutations were detected in primary lung tumor and brain metastatic tissue samples of patients with brain metastasis. *KEAP1* mutation rate was significantly higher in patients with brain metastasis than those without (P = 0.031). DNA methylation analysis revealed 15 differentially methylated blocks between primary lung tumors of patients with and without CNS metastasis. A brain metastasis risk prediction model based on these 15 differentially methylated blocks had an area under the curve of 0.94, with 87.1% sensitivity and 82.8% specificity.

**Conclusions:**

Our analyses revealed 15 differentially methylated blocks in primary lung tumor tissues, which can differentiate patients with and without CNS metastasis. These differentially methylated blocks may serve as predictive biomarkers for the risk of developing CNS metastasis in NSCLC. Additional larger studies are needed to validate the predictive value of these markers.

**Supplementary Information:**

The online version contains supplementary material available at 10.1186/s13041-021-00886-4.

## Introduction

The use of systemic targeted therapies has resulted in the prolongation of overall survival in patients with non-small cell lung cancer (NSCLC) [1]. However, earlier generations of targeted therapies are ineffective in treating intracranial disease, due to their poor penetration of the blood–brain barrier [2]; as a result, the incidence of brain metastasis is increasing.

Brain metastasis, which has a devastating impact on survival and life quality, is commonly seen in patients with NSCLC [3]. In NSCLC, brain metastases are detected at presentation in 15–26% of patients and during the disease course of about half of patients [4, 5]. Studies have reported decreased 5- and 10-year brain metastasis rates and improved 5- and 10-year disease-free survival in patients with locally advanced NSCLC after treatment with prophylactic cranial irradiation [6, 7]. Although the National Comprehensive Cancer Network recommends brain-directed screening using magnetic resonance imaging for stage II–IV NSCLC, most brain metastases are detected after symptom development, at which point very few treatment options are available [8, 9]. Radiation therapy with or without surgery is the standard treatment for patients with brain metastasis. For patients with NSCLC with brain metastasis who undergo radiation therapy, the median overall survival reaches approximately 9 months [10]. Therefore, there is a need to identify patients who have an increased risk of developing brain metastasis.

Molecular signatures for predicting brain metastasis have been investigated previously. Some studies have reported a higher incidence of brain metastasis among patients with epidermal growth factor receptor (*EGFR*)- or anaplastic lymphoma kinase (*ALK*)-mutant NSCLC [1, 11, 12]. Preclinical studies have revealed that low RNA expression levels of testis-associated actin remodeling kinase 2 (*TESK2*), selenoprotein W (*SEPW1*), and kinesin family member 16B (*KIF16B*) in pre-metastatic lung tissues are associated with a higher risk of developing brain metastasis and poorer survival [13, 14]. Moreover, the presence of metastasis-initiating tumor cells and metastatic colonization by circulating tumor cells had been found to be predictive of brain metastasis [15–17]. However, despite numerous biomarkers showing potential value for predicting brain metastasis, none of them can be used for reliable prognostication.

DNA methylation has been well established to play an important role in cancer initiation, progression, and metastasis [18–21]. Altered DNA methylation status can lead to altered gene function and cellular transformation [18–21]. Compared with genomic alterations, DNA methylation is a more promising marker for cancer screening and recurrence monitoring, and can be detected more easily [22]. Although a growing number of studies have elucidated the potential of DNA methylation as a biomarker in the screening, diagnosis, and recurrence of primary lung cancer [21, 23, 24], its utility in risk assessment for brain metastasis is largely lacking. In one study, hypomethylation and subsequent aberrant overexpression of engulfment and cell motility 3 (*ELMO3*) were found to be associated with brain metastasis [25].

In this study, we evaluated and compared the genomic and epigenomic landscapes of matched primary lung tumor tissues and brain metastatic tissues or cerebrospinal fluid, as well as primary lung tumor tissues between patients with and without central nervous system (CNS) metastases, to uncover genomic and epigenomic signatures with potential to predict brain metastasis risk. Such biomarkers can aid in identifying patients who are at a higher risk of developing brain metastasis, who may benefit from more intensive brain imaging surveillance or even prophylactic treatment.

## Methods

### Patients

A total of 29 patients with treatment-naïve NSCLC with CNS metastases who were diagnosed at the Zhejiang Cancer Hospital between January 2014 and December 2017 were retrospectively recruited. A control group comprising patients with advanced NSCLC (n = 31) who died from cancer-related causes who were not detected with CNS-related metastases nor complained of CNS-related symptoms throughout their disease course was also included. Of the 29 patients, 18 patients had brain metastasis, while the other 11 patients had leptomeningeal metastases. Brain or leptomeningeal metastases were diagnosed based on positive brain magnetic resonance imaging findings or cerebrospinal fluid (CSF) cytology results, respectively. Brain images obtained during follow-up of all patients, including the patients without CNS-related metastasis, were independently assessed by two experienced radiologists. Each patient gave their written informed consent for the use of their clinical data for research purposes at diagnosis, and the study was approved by the ethics committee of Zhejiang Cancer Hospital.

### Sample collection

**Fig. 1:**
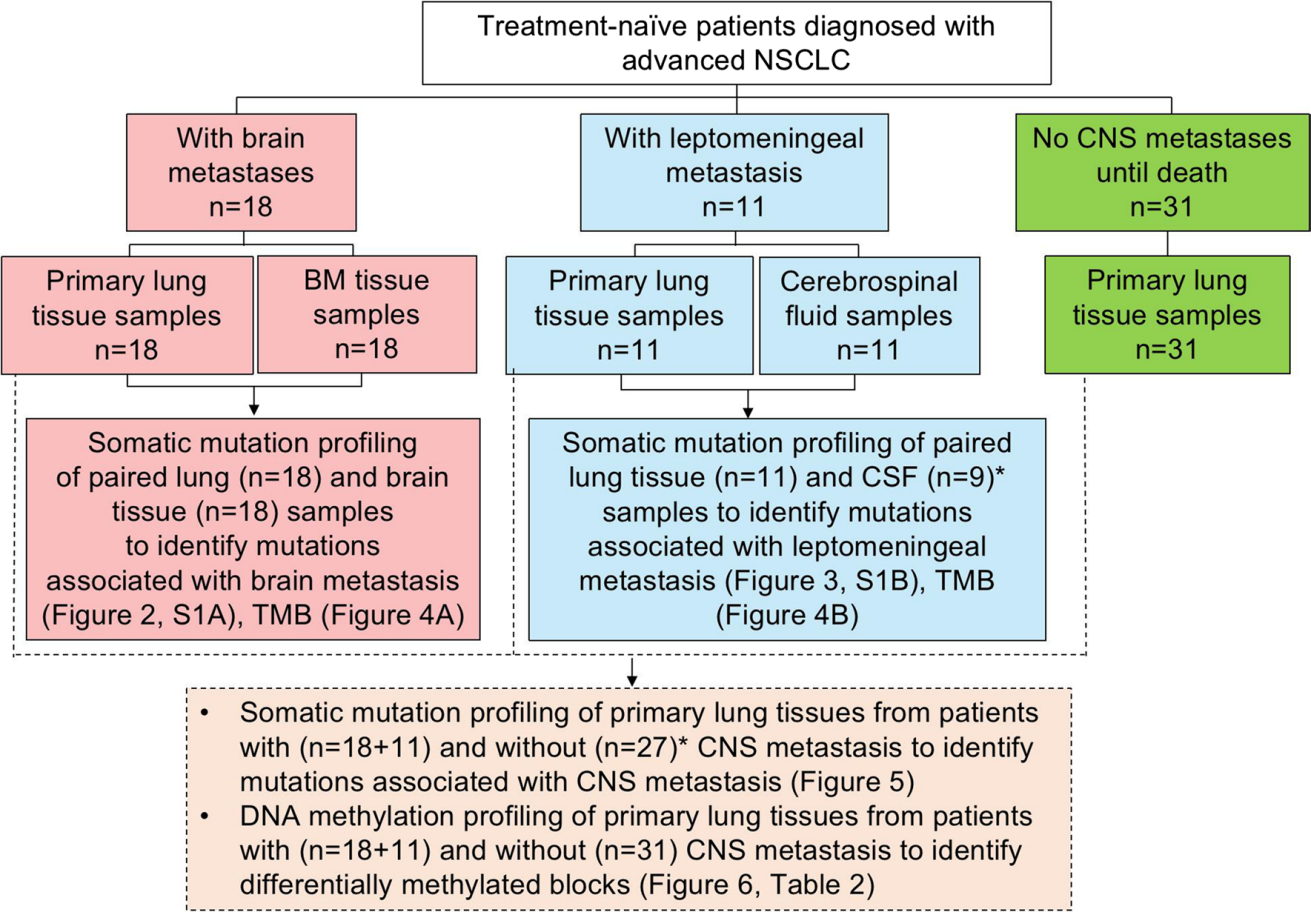
Flow chart of the study design. The study cohort included 29 patients with advanced NSCLC that metastasized to the brain parenchyma (n = 18) or leptomeninges (n = 11) and 31 patients with advanced NSCLC who never developed CNS metastasis until death. All patient samples were collected before systemic treatment and subjected to capture-based targeted genomic sequencing with a 520-gene panel and targeted bisulfite sequencing with an 80,672 CpG DNA methylation panel. Asterisks denote the 2 cerebrospinal fluid (CSF) samples and 4 lung tumor samples that failed quality control and were excluded from somatic mutation profile analysis

Matched primary lung tumor tissues and brain metastatic tissues were collected from 18 patients with brain metastasis. Matched CSF and primary lung tumor tissues were collected from 11 patients with leptomeningeal metastasis. Primary tumor tissues of 31 patients with advanced NSCLC without CNS metastases were also collected (Fig. 1). Primary lung tumor tissue samples were collected by tissue biopsy from all patients. Brain metastatic tissues were collected from 18 patients with histologically confirmed NSCLC who had undergone surgical resection of brain metastatic lesions. The histological types of each case were confirmed using patients’ electronic medical records. All primary diagnoses were further evaluated independently by two experienced pathologists according to the World Health Organization classification of NSCLC. The tissue samples needed to have at least 50% tumor cellularity. Tissue samples were stored as formalin-fixed, paraffin-embedded (FFPE) blocks. The FFPE tissue samples were subjected to targeted next-generation sequencing (NGS). For the 11 patients with leptomeningeal metastasis, approximately 8–10 mL of CSF was collected via lumbar puncture and used for cell-free DNA (cfDNA) preparation. A total of 89 paired lung tissue, brain metastatic tissue, and CSF samples were subjected to DNA isolation. However, a total of 6 samples did not pass quality control for NGS library construction; cfDNA isolated from CSF samples from 2 patients with leptomeningeal metastasis and tissue DNA isolated from primary lung tumors of 4 patients without CNS-related metastasis. Hence, only 83 samples were analyzed for somatic mutation profiles. A total of 60 primary lung tumor samples were subjected for DNA isolation and bisulfite sequencing.

### Preparation of tissue DNA and cfDNA

DNA extraction and subsequent NGS-based assays were performed by Burning Rock Biotech (Guangzhou, China). Tissue DNA was extracted using the QIAamp DNA FFPE tissue kit (Qiagen, Hilden, Germany) in adherence with the manufacturer’s instructions. An identical procedure was used to extract circulating cfDNA from CSF samples using the QIAamp Circulating Nucleic Acid kit (Qiagen, Hilden, Germany). Quantification of tissue DNA and cfDNA was performed using the Qubit 2.0 Fluorimeter with dsDNA HS assay kits (Life Technologies, Carlsbad, CA, USA). A minimum of 50 ng DNA was required for NGS library construction.

### Targeted somatic mutation sequencing

Capture-based targeted library preparation for somatic mutation profiling was carried out using a panel consisting of 520 cancer-related genes, spanning 1.7 megabases (Mb) of the human genome (OncoScreen Plus, Burning Rock Biotech, Guangzhou, China). Indexed samples were sequenced on a Nextseq500 sequencer (Illumina, San Diego, CA, USA) with paired-end reads, with an average sequencing depth of 1000 × and 10,000 × for tissue and CSF samples, respectively. Somatic mutations were called using optimized bioinformatics pipelines that can accurately report various cancer-related genetic alterations, including single-nucleotide variants (SNVs), insertion-deletion variants (indels), copy number variants (CNVs), and genomic rearrangements, as described previously [26].

### Tumor mutation burden (TMB) calculation

As described previously [27], TMB per patient was computed as a ratio between the total number of non-synonymous mutations detected and the total coding region size of the panel used using the equation below. The mutation count included non-synonymous SNVs and Indels detected within the coding region and ± 2 bp upstream or downstream region and does not include hot mutation events, CNVs, SVs, and germline single nucleotide polymorphism (SNP). Only mutations with allelic fraction (AF) of ≥ 2% for tissue samples and ≥ 0.2% for CSF samples were included in the mutation count. For accurate TMB calculation, maximum AF (maxAF) should be ≥ 5% for tissue samples and ≥ 1% for CSF samples. The total size of the coding region for estimating TMB is 1.003 Mb for the 520-gene OncoScreen Plus panel.

$$\mathrm{TMB}=\frac{\mathrm{mutation count }(\mathrm{except for CNV},\mathrm{ SV},\mathrm{ SNPs},\mathrm{ and hot mutations})}{1.003\mathrm{ Mb}}$$

### Targeted bisulfite sequencing

Bisulfite sequencing library preparation for DNA methylation profiling was performed using ELSA-seq with a panel consisting of 80,672 CpG sites, spanning 1.05 Mb of the human genome (Burning Rock Biotech, Guangzhou, China). Sequencing of target libraries was performed on a NovaSeq 6000 sequencer (Illumina, San Diego, CA, USA), with an average sequencing depth of 1000 × . DNA methylation levels were analyzed using an optimized bioinformatics pipeline based on the alignment of sequencing reads to C to T- and G to A-transformed hg19 genome, as described previously [24, 28].

### Methylation data analysis

As described previously [28], the methylation blocks were defined as the genomic region between the neighboring CpG sites, in which the r^2^ value was calculated based on a modified correlation matrix. The 80,672 CpG sites were grouped into 8312 methylation blocks using linkage disequilibrium and statistical modeling. Of the methylation blocks, 84% were annotated in genes, with 59%, 18%, and 7% in promoter regions, introns, and exons, respectively.

### Statistical analysis

Differences between groups were computed using the Fisher’s exact test for categorical data and Wilcoxon signed-rank test for continuous data. Molecular mapping of signaling pathways was performed using the Kyoto Encyclopedia of Genes and Genomes (KEGG) database. Differentially methylated blocks were identified using unpaired two-tailed Student’s *t*-test to compare the methylation profile between the no CNS-related metastasis and either brain or leptomeningeal metastasis groups. The analytical performance of the constructed model for predicting the risk of developing brain metastasis was evaluated through receiver operating characteristic (ROC) curve analysis. Statistical analyses were carried out using R software version 3.3.3 (R Foundation for Statistical Computing, Vienna, Austria), with statistical significance defined as P < 0.05.

## Results

### Baseline characteristics of patients

A total of 29 patients with NSCLC with either brain or leptomeningeal metastasis were enrolled. A total of 31 patients with stage IV NSCLC who died from cancer-related causes without developing CNS metastasis were also included as a control group. Patients in the two groups were sex and age-matched. The median age was 63 years for patients with brain metastasis, 60 years for patients with leptomeningeal metastasis, and 64.5 years for patients without CNS metastasis. For the majority of patients with CNS metastasis, the primary tumor histology was adenocarcinoma (n = 21, 72%); additionally, squamous cell carcinoma, and mixed histology accounted for four cases (14%) each. Adenocarcinoma, squamous cell carcinoma, and mixed histology accounted for 52% (n = 16), 19% (n = 6), and 29% (n = 9) of cases in the control group. Histological distribution of the cohort was not statistically significant (P = 0.064), particularly when comparing the patients with brain metastasis and patients without CNS-related metastasis (P = 0.856). All the patients without CNS metastasis had extracranial metastasis, including bone metastasis (45%), lymph node metastasis (68%), lung (35%), liver (32%), and pleura (29%). It should be noted that some patients have more than 1 organ of metastasis, which makes the percentage of all organs not equal to 100%. Patients’ detailed baseline clinical information is shown in Table 1. Figure 1 illustrates the study design.

**Table 1:** Baseline characteristics of the included patients (n = 60)

Characteristics	Brain Metastasis group (n = 18)	Leptomeningial Metastasis group (n = 11)	No Central Nervous System-related metastasis group (n = 31)
Sex, n [%]			
Male	14 [78]	7 [64]	23 [74]
Female	4 [22]	4 [36]	8 [26]
Median age (years)	63 [43–77]	60 [49–67]	64.5 [44–75]
Stage, n [%]			
IV	18 [100]	11 [100]	31 [100]
Histology, n [%]			
Adenocarcinoma	10 [56]	11 [100]	16 [52]
Squamous	4 [22]	0	6 [19]
Mixed histology	4 [22]	0	9 [29]
Presence of extracranial metastasis, n [%]			
Yes	5 [28]	10 [91]	31 [100]
No	13 [72]	1 [9]	0 [0]
Bone metastasis			
Without	16 [89]	2 [18]	17 [55]
With	2 [11]	9 [82]	14 [45]
Lymph node metastasis			
Without	18 [100]	11 [100]	10 [32]
With	0 [0]	0 [0]	21 [68]
Pleural metastasis			
Without	17 [94]	8 [73]	22 [71]
With	1 [5.6]	3 [27]	9 [29]
Lung metastasis			
Without	16 [89]	11 [100]	20 [65]
With	2 [11]	0 [0]	11 [35]
Liver metastasis			
Without	17 [94]	9 [82]	21 [68]
With	1 [5.6]	2 [18]	10 [32]
Thyroid metastasis			
Without	17 [94]	11 [100]	31 [100]
With	1 [5.6]	0 [0]	0 [0]
Adrenal gland metastasis			
Without	18 [100]	9 [82]	31 [100]
With	0 [0]	2 [18]	0 [0]
Spleen metastasis			
Without	18 [100]	10 [91]	31 [100]
With	0 [0]	1 [9.1]	0 [0]

### Somatic mutation spectra of paired primary lung tumor and brain metastatic tissue samples

**Fig. 2:**
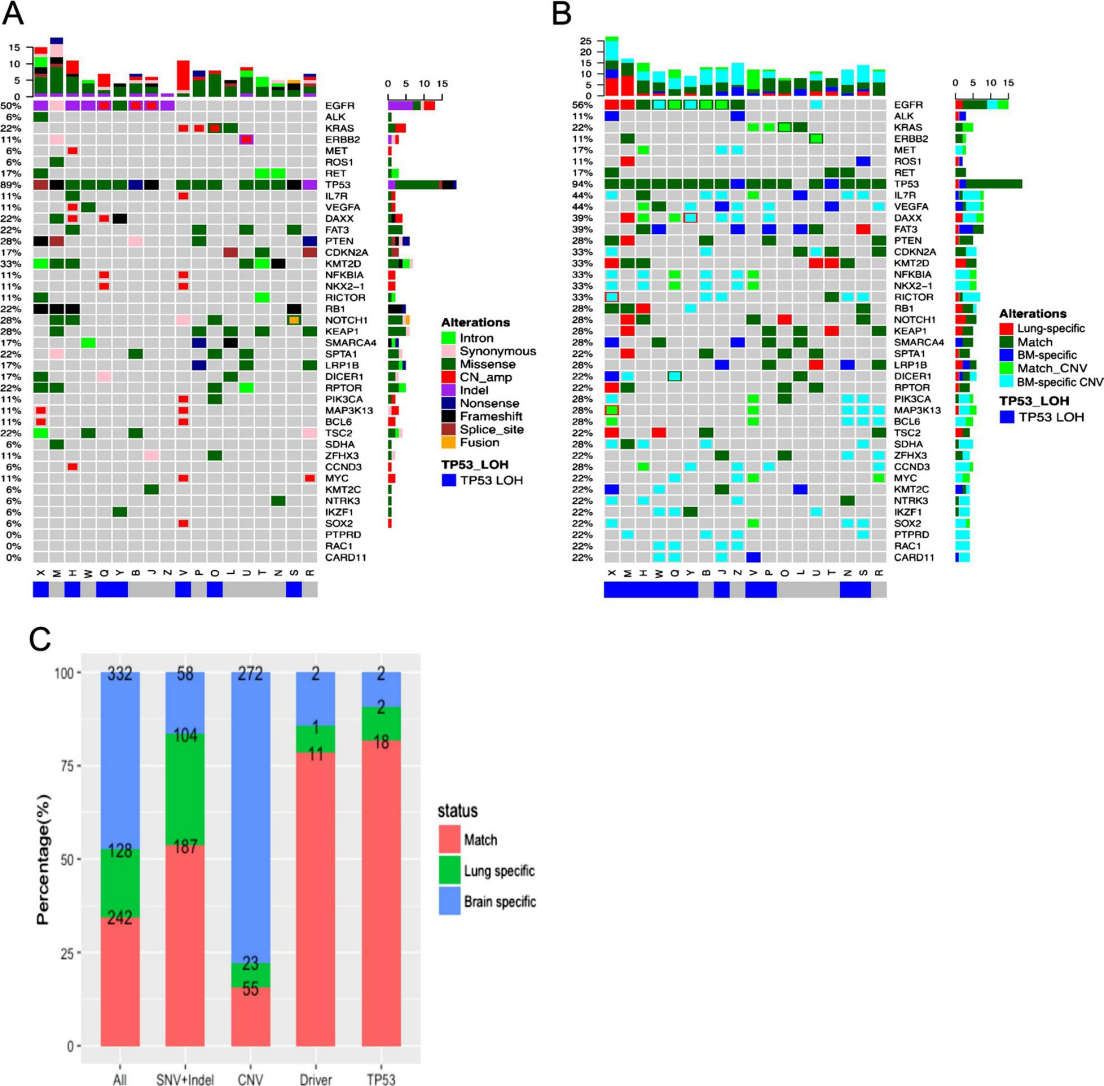
Somatic mutations in paired primary lung and brain metastatic lesions. **A** Genomic profiles of primary lung lesions (n = 18). Different colors denote different mutation types. **B** Somatic mutation profiles of paired brain metastatic lesions compared with those of primary lung lesions. Different colors denote whether mutations detected in brain metastatic tissue match with those in the paired primary lung tumor tissue (match), or were lung tumor tissue-specific or brain metastasis tissue-specific. *TP53* loss of heterozygosity (LOH) status is indicated at the bottom of the Oncoprint. The top bars summarize the number of mutations carried by a patient. The left side of the oncoprint indicates the percentage of patients with mutations in the genes indicated on the right side. The side bars summarize the total number of mutations detected and the distribution of mutation types per gene. **C** Percentages and numbers of mutations in paired primary lung and brain metastatic lesions for the indicated mutation types. *BM* brain metastatic, *TP53* tumor protein p53, *AF* allele frequency, Lung-specific, mutations only detected in primary lung lesion, *match* positive in the DNA of both primary and brain metastatic lesions, *BM-specific* mutations only detected in brain metastatic lesion, *CNVs* copy number variations, *SNVs* single-nucleotide variations, *indel* insertion-deletion variations

This analyses was performed on the somatic mutation data obtained from paired primary lung tumor and brain metastatic tissue samples of the 18 patients with brain metastasis. Somatic mutation profiling identified 370 mutations from the primary lung tumor tissue samples; of them, 291 were SNVs and indels, 78 were CNVs, and 1 was genomic rearrangement. From the brain metastatic tissue samples, 574 mutations, including 245 SNVs and indels, 327 CNVs, and 2 genomic rearrangements, were identified (Fig. 2A). The most frequently mutated gene in primary lung tumor tissue was tumor protein p53 (*TP53*), which was detected in 89% of patients, followed by *EGFR* (50%), lysine methyltransferase 2D (*KMT2D*, 33%), kelch-like ECH-associated protein (*KEAP1*, 28%), Notch receptor 1 (*NOTCH1*), phosphatase and tensin homolog (*PTEN*; 28%), and Kirsten rat sarcoma viral oncogene homolog (*KRAS*; 22%) (Fig. 2A). Further analysis revealed 242 shared mutations between the primary lung tumor and brain metastatic tissue samples, 128 primary lung tumor-specific mutations, and 332 brain metastatic tissue-specific mutations. Interestingly, the brain metastatic tissue-specific mutations were predominantly CNVs (82%, 272/332), and only 16% of the CNVs detected in brain metastatic tissue samples were also detected in the corresponding primary lung tumor samples (Fig. 2B, C). The concordance for SNVs and indels between the two types of samples was much higher than that for CNVs or rearrangements (P < 0.0001) (Fig. 2). Among the 18 cases with brain metastasis, TP53 loss of heterozygosity (LOH) was observed in the lung tumor tissue of 7 patients (39%) and in the brain metastatic tissue of 11 patients (61%). Six patients displayed TP53 LOH in both their primary lung tumor and brain metastatic tissues. The criteria for categorizing TP53 mutations as LOH include: (I) AF of TP53 > 60%; (II) AF of TP53 ≥ 1.5 times the AF of EGFR; (III) TP53 copy number loss. Additional file 2: Figure S1A summarizes all brain metastasis tissue-specific genes. Subsequent pathway analysis of these brain metastasis tissue-specific genes revealed enrichment in the phosphoinositide 3-kinase (PI3K)/protein kinase B (Akt) (P = 0.006) and focal adhesion pathways (P = 0.022, Additional file 3: Table S1).

### Somatic mutation spectra in paired primary lung tumor and CSF samples

**Fig. 3:**
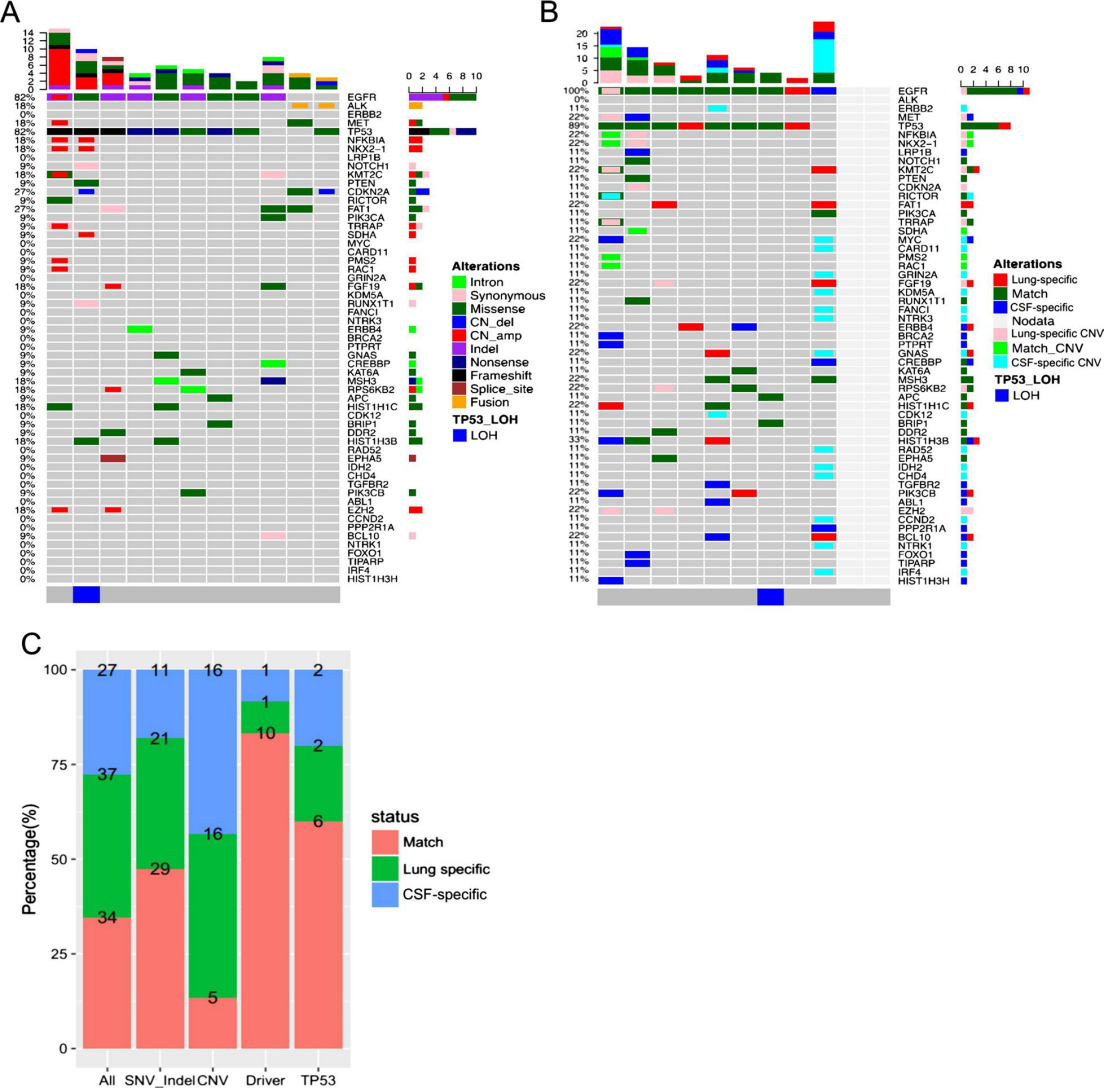
Somatic mutations in paired primary lung tumor tissue and CSF samples. **A** Genomic profiles of primary lung lesions (n = 11). Different colors denote different mutation types. **B** Genomic profiles of CSF cfDNA compared with those of primary lesions (n = 9). Two gray empty columns were the two patients who had insufficient cell-free DNA from CSF. Different colors denote whether mutations detected in CSF match with those in the paired primary lung tumor tissue (match), or were lung tumor tissue-specific or CSF-specific. *TP53* loss of heterozygosity (LOH) status is indicated at the bottom of the Oncoprint. The top bars summarize the number of mutations detected from each patient. The left side of the oncoprint indicates the percentage of patients with mutations in the genes indicated on the right side. The side bars summarize the total number of mutations detected and the distribution of mutation types per gene. **C** Percentage and number of mutations in paired primary lung lesion and CSF samples for the indicated mutation types. CSF, cerebrospinal fluid; Lung-specific, mutations only detected in lung primary lung lesion; match, positive in the DNA of both the primary lung lesion and CSF; CSF-specific, mutations only detected in CSF; *CNVs* copy number variations, *SNVs* single-nucleotide variations; *indel* insertion-deletion variations

Next, we compared the somatic mutation profiles of paired primary lung tumor tissue and CSF samples from 11 patients with leptomeningeal metastasis. Due to the limited amount of cfDNA derived from the CSF samples of two patients, somatic mutation profiling was carried out in only nine patients with leptomeningeal metastasis. A total of 71 mutations, including 50 SNVs and indels, and 21 CNVs, were identified from 11 primary lung tumor samples. A total of 61 mutations, including 40 SNVs and indels, and 21 CNVs, were detected from 9 CSF samples. The most frequently occurring mutations in lung tumor tissues were *TP53* and *EGFR*, which were each detected in 82% of patients (Fig. 3A). The other two patients were *ALK*-positive. Both of these two patients, a 49-year-old male and a 65-year-old female, had stage IVB lung adenocarcinoma with leptomeningeal metastasis and had insufficient cfDNA isolated from their CSF samples. All of the nine patients with sufficient CSF samples had *EGFR* mutations, and 8 of them had concurrent *TP53* mutations. Among all of the mutations detected, 34 mutations were present in both primary lung tumor tissue and CSF, 37 mutations were primary lung tumor-specific, and 27 mutations were CSF-specific (Fig. 3B, C). Among the CSF-specific mutations, 59% (16/27) were CNVs, among which 13 were detected in a single patient. Further, 14% of CNVs were shared by lung lesions and CSF. The concordance of SNVs and indels between the two types of samples was much higher than that for CNV or rearrangement (P = 0.0008) (Fig. 3). One patient had TP53 LOH detected in only their lung tissue, while for another patient, TP53 LOH was detected only in the CSF sample. CSF-specific mutations, which are summarized in Additional file 2: Figure S1B, were enriched in transcriptional regulation (P < 0.001) and central carbon metabolism pathways (P < 0.001) (Additional file 3: Table S1).

### High concordance in the detection of oncogenic driver and TP53 mutations

For both brain (n = 18) and leptomeningeal (n = 9) metastasis, much higher concordances were found for classic lung cancer driver mutations (79% for brain metastasis and 83% for leptomeningeal metastasis) and *TP53* mutations (82% for brain metastasis and 60% for leptomeningeal metastasis) than other genes (P < 0.001) (Figs. 2C, 3C). Eight patients with brain metastasis had *EGFR*-activating mutations [either exon 19 deletion (19del) or L858R]. Among them, seven patients had *EGFR*-activating mutations detected from both their primary lung tumor and brain metastatic tissue samples, with 88% (7/8) concordance. Of the three cases of *MET* amplification among patients with brain metastasis, two were detected from the brain metastatic tissue samples, and the other was detected from both the primary lung tumor and brain metastatic tissue samples. *KRAS* G12X and *ERBB2* (erb-b2 receptor tyrosine kinase 2) exon 20 insertion (20ins) and amplification were shared in the paired tissues of two patients and one patient with brain metastasis, respectively. For patients with leptomeningeal metastasis, all paired samples had shared *EGFR* mutations (19del, L858R, or 20ins) except for one CSF sample and two primary lung tumor samples. Interestingly, a patient with leptomeningeal metastasis had *ERBB2* amplification detected in their CSF but not in their primary tumor tissue.

### Lung primary and CNS lesions have a comparable tumor mutational burden (TMB)

**Fig. 4:**
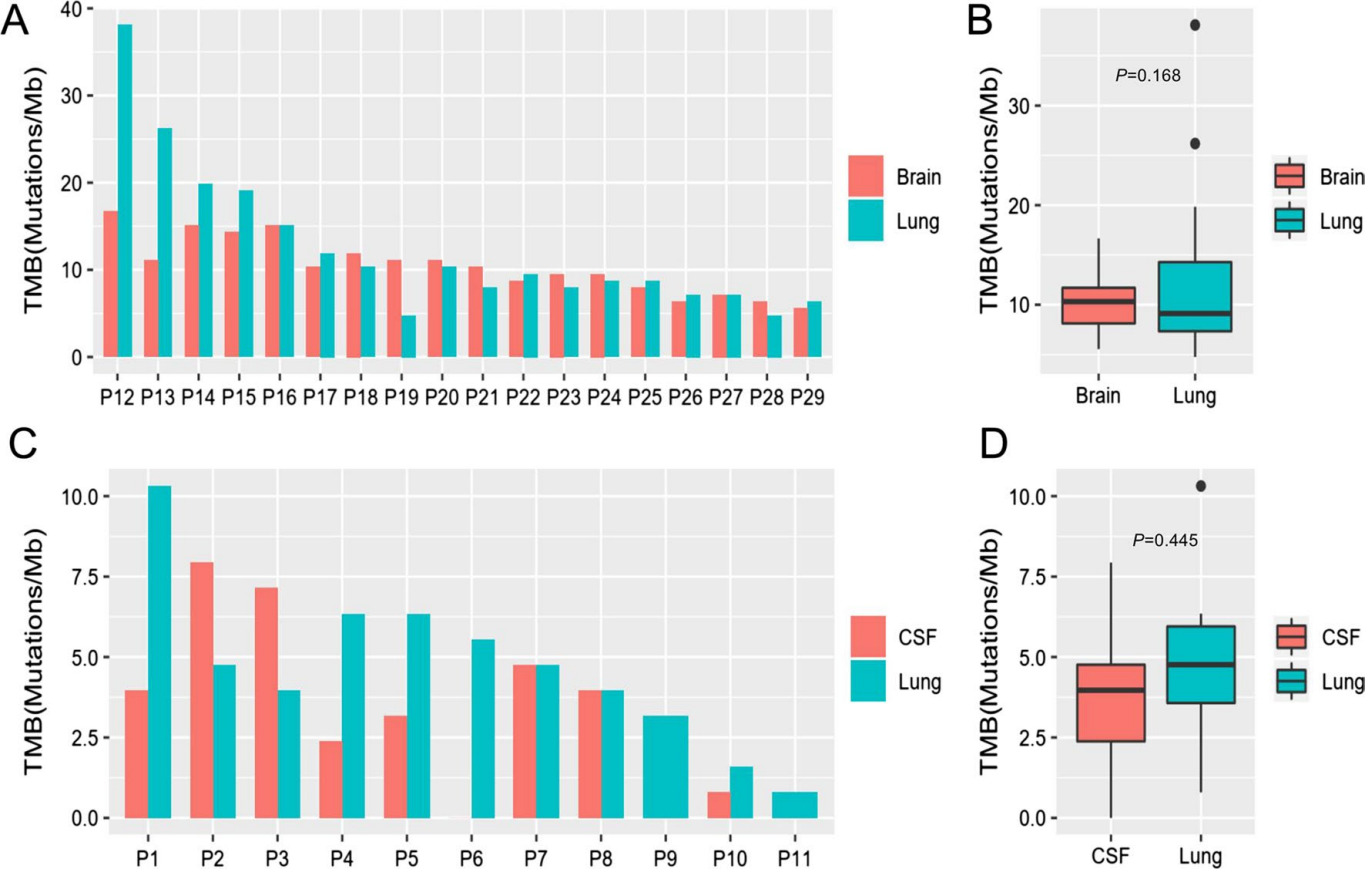
Tumor mutation burden (TMB) in paired primary lung and CNS lesions. TMB distribution of patients with brain metastasis (n = 18) (**A**, **B**) and leptomeningeal metastasis (n = 11) (**C**, **D**). **A**, **C** Histograms showing the TMB calculated for the paired primary and CNS lesions per patient. **B**, **D** Box plots comparing the TMB between primary lung lesion and brain metastatic tissues (**B**) and between primary lung lesion and CSF (**D**). P value was calculated by Wilcoxon test. *CNS* central nervous system, *BM* brain metastasis, *LM* leptomeningeal metastasis, *CSF* cerebrospinal fluid

We further investigated the TMB between paired primary lung tumor and brain metastatic tissue samples (n = 18). The median TMB for primary lung tumor was 9.13 (ranging between 4.76 and 38.1) mutations/Mb and for brain metastatic tissue samples was 10.32 (ranging between 5.56 and 16.67) mutations/Mb (Fig. 4A). Lung tumor and brain metastatic tissues had a comparable TMB (P = 0.168) (Fig. 4B). A similar trend was also observed among patients with leptomeningeal metastasis (n = 9). The median TMB for the primary lung tumor tissue of patients with leptomeningeal metastasis was 4.76 (ranging between 0.79 and 10.32) mutations/Mb, and the median TMB for their corresponding CSF samples was 3.97 (ranging between 0 and 7.94) mutations/Mb (P = 0.445) (Figs. 3C,4D). Collectively, our data demonstrate that primary lung and CNS lesions have a comparable TMB.

### Somatic mutation signature cannot predict CNS metastasis

**Fig. 5:**
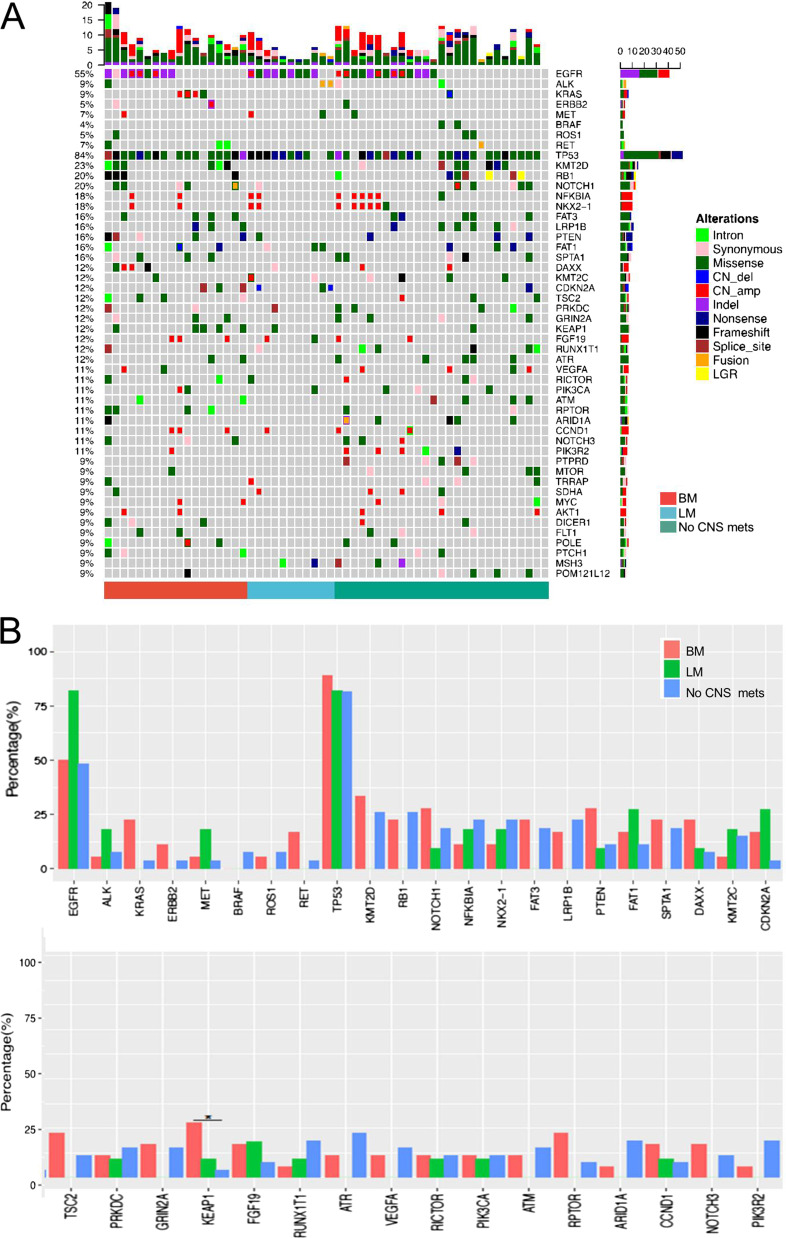
Somatic mutations of primary lesions cannot differentiate patients with and without CNS metastases. **A** Genetic profiles of primary lung lesions of patients with brain metastasis (n = 18), leptomeningeal metastasis (n = 11) or without CNS metastases (n = 27) as annotated below. Tissue DNA from four patients without CNS metastasis failed quality control and were excluded from this analysis. Different colors denote different mutation types. The top bars summarize the number of mutations detected from each patient. The left side of the oncoprint indicates the percentage of patients with mutations in the genes indicated on the right side. The side bars summarize the total number of mutations detected and the distribution of mutation types per gene. **B** Rate of detection for the indicated genes. *P < 0.05. *CNS* central nervous system, *BM* brain metastasis, *LM* leptomeningeal metastasis, no CNS metastases, patients who never developed CNS metastases before their cancer-related death; *P = 0.031 KEAP1 detected in patients with BM *vs.* those with no CNS metastases. Analyzed by Fisher’s exact test

To interrogate whether certain molecular signatures from primary lung lesions can predict the risk of developing brain metastasis, we compared the somatic mutation profiles of primary lung tumors of patients with (n = 29) and without (n = 27) CNS metastases. Tissue DNA extracted from primary lung tumor samples of four patients without CNS metastases failed quality control and were excluded from further analysis. A comparable mutation profile was derived from the primary lung tumors of the two cohorts (Fig. 5A). Figure 5B summarizes the genes with mutations occurring in four or more patients. All listed genes exhibited comparable mutation rates, with the exception of *KEAP1*, which was more frequently mutated in patients with brain metastasis (P = 0.031). Thus, somatic mutation profiles of primary lung lesions cannot distinguish between patients with and without CNS metastases.

### Identified DNA methylation blocks can differentiate patients with and without CNS metastases

**Fig. 6:**
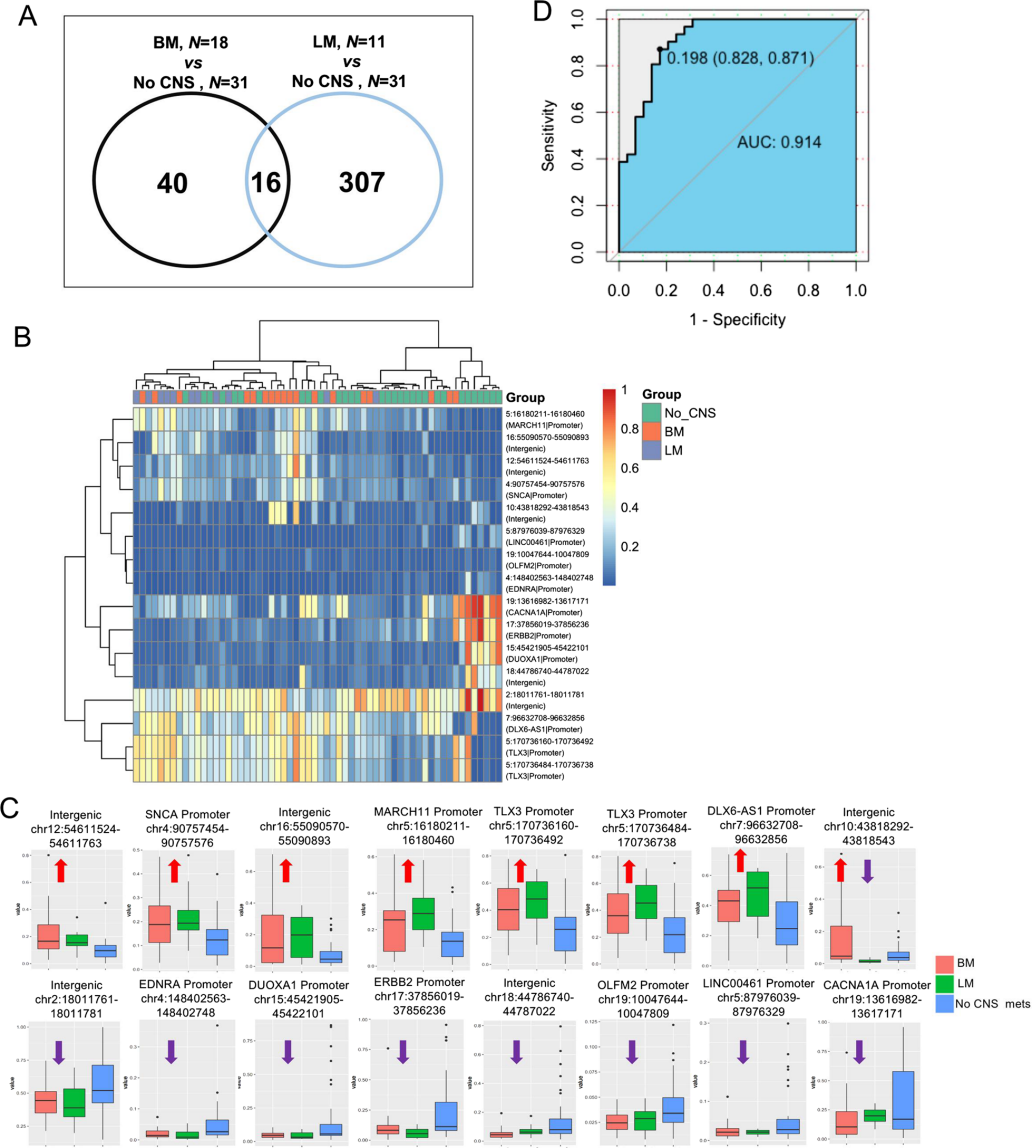
Differentially methylated blocks in patients with and without CNS metastasis. **A**–**C** 15 methylation blocks identified to be differentially methylated in primary lung tumor samples from patients with either brain or leptomeningeal metastasis as compared to patients without CNS metastasis. **A** Venn diagram showing the 16 differentially methylated blocks shared between patients with either brain or leptomeningeal metastases. **B** Heat map summarizing the unsupervised hierarchical clustering of methylation profiles of each patient for the 16 methylation markers consistently identified to be differentially methylated in primary lung lesions of patients with either brain or leptomeningeal metastasis. Differentially methylated blocks were identified using unpaired two-tailed *t*-tests with P < 0.05. **C** Box plots plotting the methylation values (y-axis) and showing the hypermethylation (red arrow pointing upwards) or hypomethylation (purple arrow pointing downwards) patterns for the 16 DNA methylation markers of the cohort. Fifteen blocks have identical patterns in patients with either brain or leptomeningeal metastases; 1 block is hypermethylated in brain metastasis but hypomethylated in leptomeningeal metastases. **D** Receiver Operating Characteristic curves were generated to assess the performance of the predictive model using the 15 methylation markers to distinguish between patients with and without CNS metastasis. *CNS* central nervous system, *BM* brain metastases, *LM* leptomeningeal metastases, no CNS metastasis, patients who never developed CNS metastasis before their cancer-related death, *AUC* area under curve

Next, we probed the DNA methylation profiles of primary lung tumor tissues obtained from patients with (n = 29) and without (n = 31) CNS metastases. As compared with the methylation status of primary lung tumor samples from patients without CNS metastasis, a total of 56 methylation blocks were identified as being differentially methylated (P < 0.05) in primary lung tumor samples from patients with brain metastasis, and 323 differentially methylated blocks (P < 0.05) were identified in primary lung tumor samples from patients with leptomeningeal metastasis (Fig. 6A). The raw methylation values for these 363 methylation blocks found to be differentially methylated in primary lung tumors of patients with brain metastasis or leptomeningeal metastasis were provided as Additional file 1: Data S1. There were more differentially methylated blocks identified in the primary lung tumor samples from patients with leptomeningeal metastasis; however, we focused on the 16 differentially methylated blocks shared between the patients with brain and leptomeningeal metastasis to identify methylation markers for CNS metastasis (Fig. 6A, B). Of these 16 differentially methylated blocks, the DNA methylation levels of seven blocks were significantly hypermethylated in patients with brain and leptomeningeal metastasis, and those of 8 blocks were significantly hypomethylated in patients with brain and leptomeningeal metastasis. One block in an intergenic region in chromosome 10 that was hypermethylated in patients with brain metastasis and hypomethylated in patients with leptomeningeal metastasis was excluded from subsequent analysis (Fig. 6B, C). Of the 15 remaining differentially methylated blocks, four blocks spanned intergenic regions and 11 covered the gene promoters of *SNCA*, *MARCH11*, *TLX3*, *DLX6-AS1*, *EDNRA*, *ERBB2*, *DUOXA1*, *OLFM2*, *LINC00461*, and *CACNA1A* (Table 2). Next, we evaluated the performance of the prediction model based on the 15 differentially methylated blocks in distinguishing patients with and without CNS metastasis. The ROC curve yielded an area under the curve of 0.941, with a sensitivity and specificity of 87.1% and 82.8%, respectively (Fig. 6D). Taken together, we identified 15 DNA methylation blocks from primary lung tumor samples that can potentially distinguish patients with and without CNS metastasis. However, it should be noted that these analyses were performed only in primary lung tumor samples and not validated in brain metastatic tissue samples from these patients.

**Table 2:** Details on the 15 differentially methylated blocks shared between patients with brain metastasis [BM) and leptomeningeal metastasis [LM)

Chromosome number	Start	End	Gene annotation	BM vs No CNS			LM vs No CNS		
				Methylation status	P value	T statistic	Methylation status	P value	T statistic
4	90757454	90757576	SNCA |Promoter	Hyper	0.034	2.24	Hyper	0.035	2.34
5	16180211	16180460	MARCH11|Promoter	Hyper	0.043	2.13	Hyper	0.007	3.13
5	170736160	170736492	TLX3|Promoter	Hyper	0.016	2.52	Hyper	0.005	3.12
5	170736484	170736738	TLX3|Promoter	Hyper	0.026	2.34	Hyper	0.003	3.33
7	96632708	96632856	DLX6-AS1|Promoter	Hyper	0.022	2.39	Hyper	0.014	2.71
12	54611524	54611763	Intergenic	Hyper	0.018	2.58	Hyper	0.028	2.39
16	55090570	55090893	Intergenic	Hyper	0.042	2.17	Hyper	0.036	2.34
2	18011761	18011781	Intergenic	Hypo	0.028	− 2.27	Hypo	0.025	− 2.40
4	148402563	148402748	EDNRA|Promoter	Hypo	0.032	− 2.23	Hypo	0.020	− 2.42
5	87976039	87976329	LINC00461|Promoter	Hypo	0.042	− 2.10	Hypo	0.010	− 2.76
15	45421905	45422101	DUOXA1|Promoter	Hypo	0.007	− 2.91	Hypo	0.005	− 2.99
17	37856019	37856236	ERBB2|Promoter	Hypo	0.036	− 2.16	Hypo	0.000	− 3.87
18	44786740	44787022	Intergenic	Hypo	0.004	− 3.13	Hypo	0.014	− 2.58
19	10047644	10047809	OLFM2|Promoter	Hypo	0.004	− 3.03	Hypo	0.022	− 2.39
19	13616982	13617171	CACNA1A|Promoter	Hypo	0.029	− 2.25	Hypo	0.024	− 2.35

*no CNS* no central nervous system-related metastasis, *hypo* hypomethylated status, *hyper* hypermethylated status

## Discussion

In this study, we investigated potential biomarkers for distinguishing patients with and without CNS metastasis based on somatic mutation and DNA methylation profiles obtained from primary lung tumor tissues. Of the 520 genes assayed for somatic mutations, only *KEAP1* was differentially mutated in patients with brain metastasis (P = 0.031). Furthermore, our study identified 15 differentially methylated blocks, which may serve as a predictive signature for CNS metastases in patients with advanced NSCLC.

Despite the advances made in the treatment of NSCLC, the prognosis of patients with brain metastasis remains dismal, partly due to the blood–brain barrier limiting the penetrability of systemic treatments. Prophylactic cranial irradiation has been shown to reduce the incidence and delay the onset of brain metastasis in patients with advanced NSCLC in several randomized studies [6, 7, 29, 30]. Multiple studies have attempted to elucidate the clinical features associated with brain metastasis that may aid in identifying patients who are at a higher risk of developing brain metastasis. A higher brain metastasis rate were found to be associated with certain clinical features, including early disease onset (diagnosis before the age of 60), non-squamous cell carcinoma histology, and the presence of bulky mediastinal lymph nodes (> 2 cm) [31, 32]. Other studies have suggested the predictive value of programmed cell death protein 1 (PD-1), liver kinase B1 (*LKB1*), *KRAS*, and *EGFR* mutations, as well as *ALK* rearrangements; however, the involvement of such mutations in the development of brain metastasis remains controversial [33–35]. In this study, we attempted to identify biomarkers predictive of brain metastasis risk at both the genomic and epigenomic levels. The genomic profiles of primary lung tumor tissues from patients with and without CNS metastasis were largely similar, including the occurrence of *EGFR*, *ALK*, and *KRAS* mutations, which have been reported to be more prevalent in patients with CNS metastasis. Of the 520 genes assayed, only the *KEAP1* mutation rate was found to be significantly higher in patients with brain metastasis, which suggests that somatic mutation profiles may not distinguish patients with and without CNS metastasis.

The search for DNA methylation-based biomarkers has been the focus of research on cancer screening, detection, and recurrence monitoring. In this study, using a fixed targeted methylation panel comprising 80,672 CpG sites, which were grouped into 8.312 methylation blocks and validated to be differentially methylated in primary lung tumor tissues (n = 48) as compared to normal lung tissues (n = 20) [28]. Among these 8312 lung cancer-specific methylation blocks, we revealed 15 genomic regions with differential methylation levels in patients with and without CNS metastasis, suggesting their potential to serve as markers for identifying patients who are at an increased risk of developing CNS metastases. Furthermore, among the differentially methylated blocks identified, 1 block covered the gene promoter of *ERBB2*, which is closely related to lung cancer and whose loss of function affects the expression of cell cycle and pro-apoptotic genes [36]. It is interesting to note that the 15 genomic regions with differential methylation levels in patients with and without CNS metastasis do not belong to pathways classically involved in both high [37] and low grade [38] brain tumors. This could also suggest their use as biomarkers capable of tracing tumor burden, especially if they were also altered in the brain metastasis of lung tumors. Hence, extensive studies on these 15 genomic regions are warranted.

Due to branched evolution, intracranial lesions and primary lung lesions demonstrate genetic heterogeneity [39–41]. However, research to compare the genomic profiles of brain metastatic lesions and primary lung lesions is extremely limited, primarily due to the challenge of obtaining brain metastatic tissues. Most of the comparative studies to date have utilized CSF instead of brain metastatic tissues. Our data revealed distinctive mutation profiles between primary lung tumor and brain metastatic tissues, including an abundance of unique CNVs in brain metastatic tissues. Studies have shown that genomic instability at the chromosomal and mutational levels may result from an increased number of CNVs [42]. In agreement with published studies, our study also revealed multiple brain metastasis-specific CNVs, which suggests their importance in the development of brain metastasis. Such CNVs may be the products of tumor evolution or may even act as key mutations, which are predominant in brain metastasis [26]. Collectively, our data imply that brain metastasis-specific CNVs may be critical in the metastasis of lung cancer to the brain.

Our study has a few limitations. First, it was a retrospective study, which limited the data that we could analyze. Second, samples were obtained from a single center, which limited the number of patient samples that can be included in the study. Our findings were also limited by the use of a fixed panels that only included 520 cancer-related genes for somatic mutation profiling and 80,672 CpG sites for DNA methylation profiling of our samples. It is possible that some mutations in genes not included in the gene panel and DNA methylation markers in genomic regions farther from the CpG islands were missed; hence, whole exome sequencing or whole genome bisulfite sequencing of primary lung samples from patients with or without CNS could be explored to comprehensively identify novel markers. Furthermore, the 15 DNA methylation markers were only identified to be differentially methylated in the primary lung tumors samples and were not validated in brain metastatic tissues, CSF, or blood samples, which could limit its clinical application, particularly for patients with limited lung tissue samples. However, these 15 genomic regions were identified from the 8312 lung cancer-specific methylation blocks, which suggests the biological relevance of these regions and deserves further investigation. Large multi-center studies are therefore needed to validate our results and confirm the predictive value of the 15 DNA methylation markers we have identified in our study.

In conclusion, we have uncovered a set of differentially methylated blocks that can potentially predict the development of CNS metastasis. This study has provided evidence that patients with advanced NSCLC can be stratified according to their likelihood of developing brain metastasis using DNA methylation profiling. Our findings may facilitate the implementation of a prevention strategy for patients who carry a higher risk of developing brain metastasis.

## Supplementary Information

**Additional file 1: Data S1.** Raw methylation values for 363 blocks.

**Additional file 2: Figure S1.** Brain-specific mutations.

**Additional file 3: Table S1.** List of genes and signaling pathways identified as brain metastatic tissue (BM)-specific or cerebrospinal fluid (CSF)-specific.

## Data Availability

The methylation data for all patients were included as Additional file 1: Data S1. All the other data included in this study are available upon request by contact with the corresponding author.

## Abbreviations

cfDNA: Cell-free DNA
CNS: Central nervous system
CNV: Copy number variations
CSF: Cerebrospinal fluid
Indels: Small insertion-deletion variations
FFPE: Formalin-fixed, paraffin-embedded
LOH: Loss of heterozygosity
Mb: Megabases
NGS: Next-generation sequencing
NSCLC: Non-small-cell lung cancer
ROC: Receiver operating characteristic
SNV: Single nucleotide variations
TMB: Tumor mutation burden
