# Supplementary material for: Integrated genomic and DNA methylation analysis of patients with advanced non-small cell lung cancer with brain metastases
Source: Mol Brain. 2021 Dec 24;14:176. doi: 10.1186/s13041-021-00886-4 (PMC8710019; doi:10.1186/s13041-021-00886-4)
Supplement: Supplementary file 2 — Additional file 2: Figure S1. Brain-specific mutations. [file 13041_2021_886_MOESM2_ESM.pptx]

## Slide 1
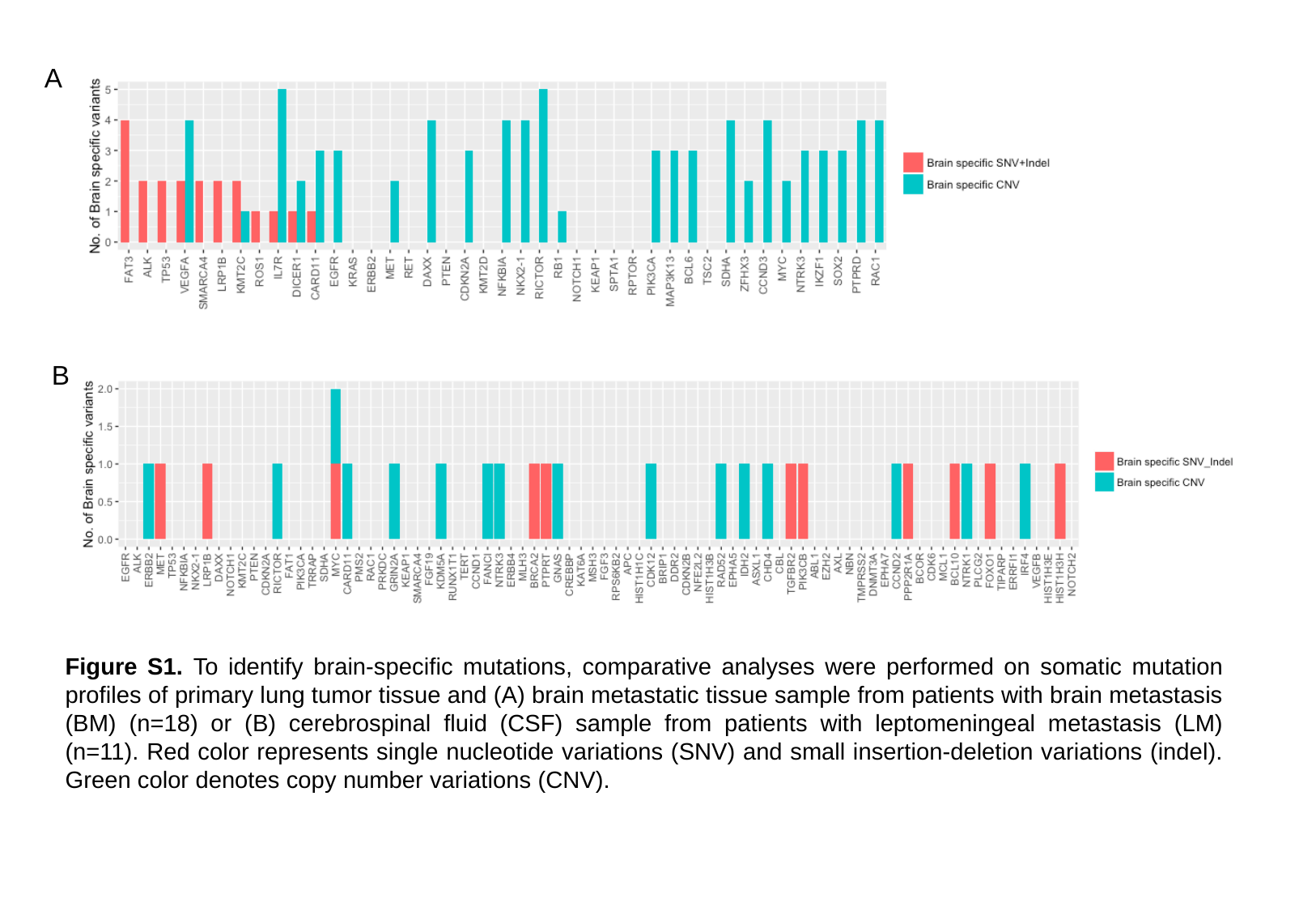

A
B
Figure S1. To identify brain-specific mutations, comparative analyses were performed on somatic mutation profiles of primary lung tumor tissue and (A) brain metastatic tissue sample from patients with brain metastasis (BM) (n=18) or (B) cerebrospinal fluid (CSF) sample from patients with leptomeningeal metastasis (LM) (n=11). Red color represents single nucleotide variations (SNV) and small insertion-deletion variations (indel). Green color denotes copy number variations (CNV).
