# Supplementary material for: Integrated genomic and DNA methylation analysis of patients with advanced non-small cell lung cancer with brain metastases
Source: Mol Brain. 2021 Dec 24;14:176. doi: 10.1186/s13041-021-00886-4 (PMC8710019; doi:10.1186/s13041-021-00886-4)
Supplement: Supplementary file 3 — Additional file 3: Table S1. List of genes and signaling pathways identified as brain metastatic tissue (BM)-specific or cerebrospinal fluid (CSF)-specific. [file 13041_2021_886_MOESM3_ESM.docx]

**Table S1**. List of genes and signaling pathways identified as brain metastatic tissue (BM)-specific or cerebrospinal fluid (CSF)-specific

|  | KEGG terms | Genes involved in the pathway | P-value |
| --- | --- | --- | --- |
| BM-specific | hsa04151:PI3K-Akt signaling pathway | *CCND3, VEGFA, RAC1, IL7R* | 0.006 |
|  | hsa04510:Focal adhesion | *CCND3, VEGFA, RAC1* | 0.022 |
| CSF-specific | hsa05230:Central carbon metabolism in cancer | *NTRK3, PIK3CB, NTRK1, ERBB2, MET, MYC* | 1.12E-04 |
|  | hsa05202:Transcriptional misregulation in cancer | *CCND2, NTRK1, TGFBR2, MET, FOXO1, MYC, HIST1H3H* | 3.69E-04 |
